# Coal Gangue Ecological Matrix Coupled with Microalgae for Soil Improvement and Plant Growth in Reclaimed Mining Areas

**DOI:** 10.3390/biology14070741

**Published:** 2025-06-21

**Authors:** Shuyu Yu, Jinning Li, Dandan Du, Hao Li, Jiayong Hao, Zedong Teng, Xiang Ji

**Affiliations:** 1 College of Water Conservancy and Civil Engineering, Inner Mongolia Agricultural University, Hohhot 010018, China; yushuyu@imau.edu.cn (S.Y.); duddpublic@163.com (D.D.); hao.li@imau.edu.cn (H.L.); 2 College of Life Sciences, Inner Mongolia Agricultural University, Hohhot 010010, China; ruby03200@163.com; 3 Ordos Dongsheng District Environmental Protection Management Co., Ltd., Ordos 017000, China; environmental_hjy@163.com; 4 School of Energy and Environmental Engineering, University of Science and Technology Beijing, Beijing 100083, China; 5 College of Agriculture, Chifeng University, Chifeng 024000, China

**Keywords:** coal gangue, microalgae, coupling effect, mining area reclamation, soil improvement

## Abstract

Coal gangue, a primary byproduct of coal mining, causes significant environmental harm due to its improper utilization. This research proposes integrating microalgae with coal gangue-derived ecological products to improve soil conditions in ecologically vulnerable coal-mining regions. A field-scale experiment at the Jintong Coal Mine tested soils amended with varying proportions of a coal gangue ecological matrix (0%, 10%, 30%, and 50%), with and without microalgae inoculation. The results demonstrated that coal gangue addition caused undesirable soil pH decreases (11.30~42.20%) while increasing total dissolved solids (506.88~524.93%) and organic matter (8.51~46.81%). These effects were mitigated by the presence of microalgae. Microalgae play a role in regulating soil nutrient profiles, enhancing enzymatic activities, and modulating the microbial community structure. For example, they restored catalase activity under the stress imposed by coal gangue and stimulated urease activity at higher coal gangue proportions. Plant growth trials revealed that adding 30% coal gangue or combining coal gangue with microalgae significantly promoted the growth of *Medicago sativa* L. In summary, coupling the coal gangue ecological matrix with microalgae effectively enhances soil quality. Maintaining the coal gangue addition at 30% or less in conjunction with microalgae application represents an optimal approach for soil improvement in mining areas.

## Simple Summary

A new technology integrating coal gangue-based ecological materials with microalgae was developed to improve reclaimed soil in ecologically fragile mining areas. Following one year of continuous soil amelioration practices, comprehensive analyses revealed significant improvements in the three key soil parameters of nutrients, enzyme activity, and microbial community structure. Furthermore, cultivated *Medicago sativa* L. exhibited optimal growth performance, which collectively indicates successful ecological restoration of the edaphic environment. This study provides a sustainable solution for ecological restoration in coal-mining areas.

## 1. Introduction

The ecologically vulnerable mining regions in western China have evolved into strategically significant coal production bases under national energy policies. Approximately 40% of global coal production relies on open-pit mining due to a combination of geological, economic, and technological factors [1]. Advances in equipment and automation have enhanced efficiency, though environmental concerns (e.g., land degradation and water pollution) sometimes restrict use in regions with stricter regulations (e.g., parts of Europe). Notably, leading coal-producing nations, including China, India, Indonesia, and Australia, predominantly rely on open-pit mining techniques owing to a confluence of geological endowments and policy-driven advantages [2]. Therefore, this mining method has also led to irreversible degradation of ecosystem functions, especially manifested through intensified soil erosion, depletion of groundwater levels, and reduction of biodiversity, thereby damaging the basic ecological security of these geologically sensitive areas [3].

Contemporary investigations into open-pit coal mine reclamation have undergone a paradigm shift, transitioning from monodimensional land rehabilitation practices to interdisciplinary frameworks that synergistically integrate pedological reconstruction, biodiversity rehabilitation, hydrogeological cycle restoration, and socioeconomic revitalization [4]. As a pivotal scientific endeavor in land reclamation and ecological rehabilitation, soil reconstruction serves not only as the foundation for restoring anthropogenically disturbed pedogenic processes but also governs the long-term efficacy of ecosystem services through its decisive influence on reconstructed soil’s structural integrity (e.g., porosity hierarchy and aggregate stability) and functional capacity (e.g., nutrient cycling and microbial metabolic activity) [5].The restoration and improvement of reclaimed soil is one of the most effective measures for re-establishing damaged ecosystems in mining areas, and can also effectively improve the soil environment of abandoned mining areas [6]. Recent studies have empirically validated the efficacy of employing locally derived waste materials from mining operations as strategic amendments for ecological restoration and soil rehabilitation initiatives [7]. This approach not only demonstrates sustainable resource circularity within extractive industry contexts but also addresses critical challenges in the remediation of degraded terrestrial ecosystems through the beneficial reuse of indigenous geological byproducts. Contemporary research further substantiates that such geo-sourced amendments enhance pedogenic processes, improve soil structural integrity, and facilitate the re-establishment of biogeochemical cycles in anthropogenically disturbed landscapes.

Coal gangue, a carbonaceous solid byproduct generated during coal mining and beneficiation processes, has progressively accumulated as a significant industrial residue at extraction sites across China [8]. However, the long-term accumulation of a large amount of coal gangue not only occupies valuable land resources (forming over 2600 gangue mountains), but also poses multiple threats to the environment and ecosystem. Therefore, the comprehensive utilization of coal gangue has become an important way to promote the green transformation of the coal industry and achieve sustainable development. Coal gangue exhibits a consolidated texture dominated by phyllosilicate assemblages, predominantly comprising clay minerals (e.g., illite and kaolinite), chlorite group minerals, and amorphous organic matter. The accessory constituents, accounting for a relatively small proportion, include detrital quartz (α-SiO_2_) and authigenic pyrite (FeS_2_). Geochemically, the bulk composition is characterized by silico-aluminous dominance, with SiO_2_ (40–55 wt%) and Al_2_O_3_ (25–35 wt%) collectively constituting over 70% of the total oxide content [9]. The mineral compositions of coal gangue are similar to that of natural soil, and its improvement potential and ecological restoration effect have been achieved in practice. The main utilization methods include direct covering, mixing with other substrates, and modified treatment before use [10].

However, the direct use of coal gangue as a substrate can result in nutrient deficiencies. Moreover, adding organic matter (e.g., coal combustion byproducts, sludge, and wood chips) to the soil can improve soil fertility and accelerate ecosystem recovery [11]. Therefore, by optimizing the utilization of coal gangue, it is also possible to improve the soil conditions in mining areas, thereby significantly enhancing the potential for vegetation restoration. Some studies have also shown that microorganisms, such as phosphorus-solubilizing bacteria [12] and sulfate-reducing bacteria [13], can be used to overflow the favorable components of the coal gangue. Microalgae, similar to microorganisms, also have physiological and ecological functions such as nitrogen fixation, carbon fixation, sugar secretion, and phosphorus solubilization [14]. Microalgae can control many pollutants which are underlying factors for soil deterioration, such as heavy metals, pesticides, antibiotics, and microplastics, with their particular cell structure or functional group. In addition, as primary producers, algae can improve soil fertility through photosynthesis and nitrogen fixation [15]. However, currently, in ecologically fragile mining areas, there is a lack of research on the coupling of a coal gangue-based ecological matrix and microalgae for soil restoration and improvement, which hinders the promotion and application of this ecological strategy.

This study aims to address these gaps using the following methods: evaluating the performance of integrated remediation strategies (e.g., the reuse and repair of waste based functional materials, algae activators enhancing soil vitality, and plant growing) in typical coal mining areas of Western China; and exploring the interrelationships among soil physicochemical properties, enzyme activities, and microbial community structure during the implementation of remediation and improvement strategies. Coal gangue and microalgae may play an important role in improving soil ecology. The results of this study can provide a unique insight for the resource utilization and eco-friendly management of coal gangue.

## 2. Materials and Methods

### 2.1. Experimental Materials and Chemicals

**Figure 1:**
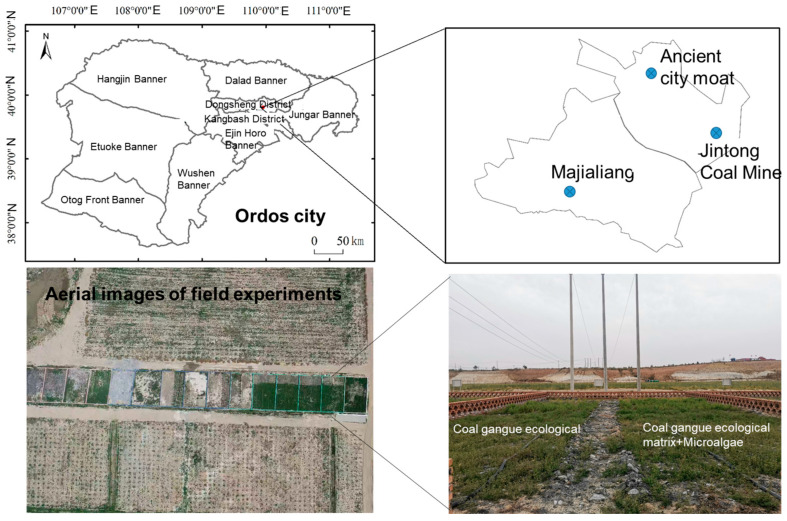
Location map of the Jintong Coal Mine and field experimental plot.

The coal gangue utilized in this study was procured from Jintong Coal Mine, which is located in the mining area shown in Figure 1. Physicochemical characterization revealed the substrate exhibited a circumneutral pH profile (mean value = 7.41) with moderate organic matter content (310.67 ± 162.69 mg kg^−1^). The microalgal inoculum, consisting of nitrogen-fixing photosynthetic algal consortia (Chlorophyta–Cyanobacteria symbionts) suspended in purified aqueous medium, was commercially sourced from Inner Mongolia Arge Life Science Co., Ltd. (Tongliao, China), with a guaranteed cellular density of ≥1.0 × 10^6^ cells mL^−1^. Analytical-grade chemical reagents (Aladdin Reagent Co., Ltd., Shanghai, China) and ultrapure water (18.2 MΩ·cm resistivity) were employed throughout experimental procedures to ensure methodological rigor. All materials underwent quality verification protocols prior to application, including spectral purity confirmation for solvents and sterility validation for biological components.

### 2.2. Field Experiment Design

The study was conducted in the Jintong Coal Mine in Ordos city (39°00′ N~40°00′ N; 109°00′ E~110°00′ E), China, in the Central Inner Mongolia Autonomous Region (Figure 1). This region belongs to a typical temperate continental climate with four distinct seasons. The annual average temperature ranges from 5.3 to 8.7 °C, with the coldest month of January having an average temperature between −10 and −13 °C, and the hottest month of July having an average temperature between 21 and 25 °C. The average precipitation is between 300 and 400 mm, and the annual precipitation is concentrated from July to September. The evaporation rate is large, with an annual evaporation rate of 2000–3000 mm. Inner Mongolia is the main production area for *Medicago sativa* L. seeds and grass in China, and also a major utilization area. Planting *Medicago sativa* L. can provide high-quality forage for the development of animal husbandry in Inner Mongolia.

The experimental site comprised a total area of 2 acres (0.80 hectares), systematically subdivided into four equal-sized rectangular plots (17 m × 20 m each) arranged in a randomized complete block design. Among them, 1/4 of the area inside each block was planted with *Medicago sativa* L. This partitioning strategy ensured homogeneous soil characteristics across all treatment units (0.20 ha/plot) while maintaining necessary buffer zones between adjacent plots to prevent edge effect interference. The pH of soil in the restoration area was 8.27 ± 0.02, the organic matter content was 8.37 ± 1.90 mg/kg, and the available phosphorus content was 5.76 ± 0.44 mg/kg.

In order to evaluate the improvement effect of different measures on reclaimed soil, four different treatments were designed: (1) primitive soil; (2) soil containing 10% coal gangue; (3) soil containing 30% coal gangue; and (4) soil containing 50% coal gangue. Two groups were set up for each treatment, with the addition of organic matter (0% CG, 10% CG, 30% CG, and 50% CG), and organic matter and microalgae (0% CGM, 10% CGM, 30% CGM, and 50% CGM), respectively (Table 1). Coal gangue ecological materials were directly sprayed into the soil before sowing and mechanically plowed and mixed to achieve uniform mixing. Microalgae were spread into the soil through artificial irrigation.

The planting time of *Medicago sativa* L. was May 2023. After one year of improvement, eight mixed samples were collected using the multipoint method to analyze soil physicochemical properties, enzyme activities, and the soil microbial community. Each mixed sample consisted of 5 samples from the same experimental area, collected at the intersection of the two diagonals and their midpoint in the experimental area.

### 2.3. Determination of Basic Physical and Chemical Properties of Soil

The soil pH value and total dissolved solids (TDS) were determined by preparing a 1:5 (*w*/*v*) soil-to-deionized water suspension [16]. The mixture was mechanically shaken for 1 min to ensure homogeneity, followed by a 30 min equilibration period to allow complete ion dissolution. Following equilibration, the supernatant pH was measured using a calibrated pH meter, while the TDS was quantified through the same suspension using an electrode standardized with NaCl reference solutions. Soil organic matter (OM) quantification was performed using the standardized dichromate oxidation method under strongly acidic conditions. The available phosphorus (AP) was determined using the 0.5 mol/L sodium bicarbonate extraction method coupled with the molybdenum–antimony ascorbic acid spectrophotometric colorimetric assay [17]. The available nitrogen (AN) was measured using an alkaline diffusion approach, where ammonia liberated from hydrolysis was captured in boric acid absorbent and stoichiometrically determined by titration with a certified sulfuric acid standard solution. Soil sulfate (SU) content was quantified using wavelength-dispersive X-ray fluorescence spectroscopy (WD-XRF) following standardized analytical protocols [18]. Each indicator was tested three times repeatedly to reduce errors and to verify the results.

### 2.4. Enzyme Activity Assays

Soil enzymatic activities, serving as critical biomarkers of microbial metabolic processes and bioremediation efficacy, were quantitatively analyzed through standardized spectrophotometric assays. Alkaline phosphatase (ALP) activity was determined via the p-nitrophenyl phosphate hydrolysis methodology, where enzymatic liberation of p-nitrophenol was quantified spectrophotometrically at 405 nm (UV-2600, Shimadzu, Kyoto, Japan) following 1 h incubation at 37 °C in modified universal buffer (pH 11.0). Soil catalase activity (CAT) was assayed using hydrogen peroxide decomposition kinetics, with residual H_2_O_2_ concentrations measured through titanium sulfate complex formation at 510 nm absorbance 20 min after reaction termination. Urease activity was evaluated via substrate-dependent ammonium release using urea–CaCl_2_ incubation (2 h, 37 °C), where NH_3_-N production was colorimetrically detected through indophenol blue formation at the 578 nm wavelength following the Berthelot reaction protocol [19]. Sulfate reductase activity (SR) was measured through antigen–antibody recognition using double-antibody sandwich ELISA (Human ELISA Kit, E-EL-R1084c) with optical density determination at 450 nm/630 nm dual wavelengths. Each indicator was repeated 3 times to reduce experimental errors and to verify the results.

### 2.5. Soil Microbial Community Analysis

Soil microbial community analysis was conducted through standardized molecular ecological protocols. Triplicate 5 g soil aliquots from each experimental treatment were aseptically collected following the one year recovery period and immediately preserved at −20 °C under cryogenic storage conditions to maintain nucleic acid integrity. High-throughput sequencing of 16S rRNA gene hypervariable regions (V3–V4: primer pairs “338 F (5′-ACTCCTACGGGAGCAGCAG-3′) and 806 R (5′-GGACTACHVGGGTWTCTAAT-3′)) was executed on the Illumina NovaSeq 6000 platform (2 × 250 bp paired-end strategy; Shanghai Meiji Biomedical Technology Co., Ltd., Shanghai China).

Alpha diversity analysis, Bray Curtis distance principal component analysis, and microbial community structure analysis were all conducted using testing platforms. COG functional abundance statistics in the “PICRUSt2 (v2.2.0-b)” package were used to predict gene function in microbial communities, which can reveal more detailed changes in the characteristics of functional genes by extracting abundance and functional data and visualizing them in groups. In this research, we used the analysis platform of Shanghai Meiji Biotechnology Co., Ltd. (Shanghai, China), and standardized processes such as homologous sequence alignment, numbering mapping, and functional classification, to achieve systematic annotation of protein functions. Then, we calculated the relative abundance of each protein in different treatments. Multivariate statistical integration of microbial community structure and environmental parameters was achieved through Mantel tests (999 permutations) and redundancy analysis (RDA) with Hellinger-transformed abundance data, as detailed in contemporary geomicrobiological methodologies.

### 2.6. Data Analysis

The organization and collection of data were mainly carried out using Excel spreadsheets. Plots were generated using the Origin 2025 mapping software. SPSS20.0 (SPSS Inc., Chicago, IL, USA) was used to analyze the significant differences between groups. Principal component analysis (PCA) was used to study the beta diversity patterns of the microbial community structure under different experimental treatments. The heatmap analyzed the correlation between multiple environmental variables and soil enzyme activity variables. Redundancy analysis (RDA) quantified the multivariate relationship between environmental variables and microbial community composition. The relationship between the bacterial community, soil physicochemical characteristics, and enzyme activities were assessed through the platform of Shanghai Meiji Biomedical Technology Co., Ltd.

## 3. Results and Discussion

### 3.1. Soil Physicochemical Characteristics in Field Improvement Experiments

**Figure 2:**
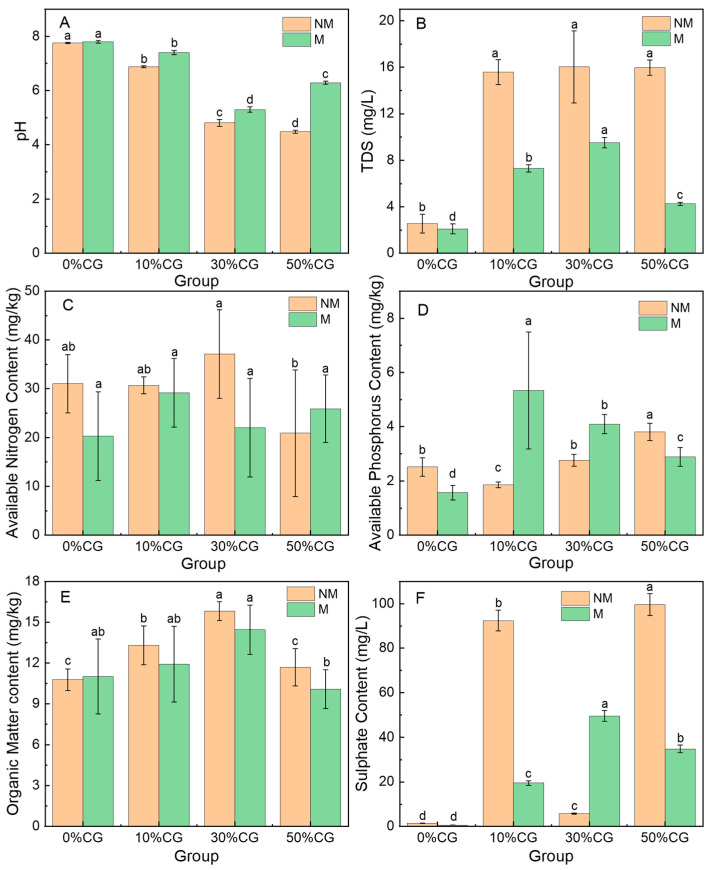
Variance of soil physicochemical properties. (**A**) pH; (**B**) TDS; (**C**) available nitrogen; (**D**) available phosphorus; (**E**) organic matter; (**F**) sulfate. NM: Group without added microalgae. M: Group with added microalgae. Error bars represent the standard deviation. Different letters indicate significant differences within the group (*p* < 0.05).

The physicochemical characteristics of soil provide vital diagnostic parameters for evaluating its biological functionality and overall health condition. The experiment quantified key soil parameters including pH, TDS, AN, AP, OM, and SU across different treatments, with the comparative results graphically represented in Figure 2. The original soil was in a relatively alkaline state, and the pH of the group without adding coal gangue was significantly higher than that of the group with added gangue (*p* < 0.05). The incorporation of coal gangue as an ecological amendment elicited a significant decrease in soil pH, reaching a maximum of 42.20%, with the magnitude of acidification demonstrating a dose-dependent relationship with the proportion of added gangue material (Figure 2A). The observed acidification likely originates from sulfur oxidation processes and acidic leachates characteristic of weathered coal gangue matrices [20]. The sulfur content in the coal gangue used in this study was 16.81 mg/kg, mainly in the form of pyrite. Notably, comparative analysis revealed significantly mitigated pH reduction in microalgae-amended treatments compared to non-amended controls under equivalent coal gangue incorporation rates, demonstrating the pH-buffering capacity of algal biomass in gangue-remediated soils. This regulatory function is attributed to microalgal exudation of alkaline metabolites and enhanced cation exchange capacity through EPS secretion [21]. Coal gangue application induced a five-times increase in soil TDS, attributable to the accelerated leaching of soluble salts (Ca^2+^, SO_4_^2−^, and Cl^−^) from gangue weathering products and enhanced ionic migration through newly formed pore networks (Figure 2B). Notably, microalgae amendment demonstrated significant mitigation efficacy against coal gangue-induced TDS accumulation in soil systems, with TDS elevation rates reduced by 47.6%~74.88% compared to gangue-only controls. Studies have also shown that the combined application of microalgae and charcoal materials can improve soil salinization, which can to some extent reduce the dissolved ion content of soil [22].

Soil available nitrogen dynamics revealed a unimodal relationship with coal gangue incorporation levels, characterized by enhancement at moderate additions (0–30%) followed by significant depletion at higher loading rates (>30%) (Figure 2C). This may be due to the imbalance of the soil carbon and nitrogen ratio caused by a high addition of coal gangue, which stimulates the proliferation of microorganisms which compete to consume available nitrogen in the soil for the synthesis of their own cell tissues, resulting in a decrease in soil available nitrogen content. Available phosphorus (AP) content in soil exhibited a biphasic response to coal gangue incorporation, characterized by an initial depletion phase followed by a subsequent elevation with escalating amendment rates, and the group with 50% coal gangue addition was significantly higher than other treatment groups (*p* < 0.05). This reveals complex geochemical interactions (coal gangue minerals significantly alter the pH and Eh environment of soil through processes such as oxidation and dissolution, while introducing highly reactive ions such as iron, aluminum, and calcium, thereby promoting the fixation and release of phosphorus) between gangue-derived minerals and soil phosphorus speciation (Figure 2D). Microalgae amendment induced a significant reduction in AN content, while simultaneously elevating available AP levels [23,24]. This bidirectional regulatory effect likely stems from the following: (1) preferential nitrogen assimilation by microalgae for photosynthetic biomass production, depleting bioavailable nitrogen pools; (2) algal exudation of phosphatases and low-molecular-weight organic acids (e.g., citric acid and oxalic acid) that promote mineral phosphorus solubilization through ligand–exchange reactions [25]; and (3) microalgae-mediated shifts in microbial community function that enhance phosphorus mineralization while exacerbating nitrogen immobilization [26], primarily driven by elevated carbon-to-nitrogen (C/N) ratios of algal-derived organic substrates [27].

The organic matter content of the group with a 30% CG addition was significantly higher than that of all other treatments. After adding microalgae, it was only significantly higher than the group with a 50% CG addition, and there was no significant difference compared to other groups (*p* < 0.05). The soil OM content exhibits a biphasic response characterized by an initial increase followed by a gradual decline with incremental coal gangue application, indicating a concentration-dependent threshold effect (Figure 2E). This non-monotonic pattern likely arises from the dynamic interplay between organic carbon enrichment mechanisms (e.g., exogenous carbon input and enhanced microbial humification) and subsequent inhibitory processes (e.g., heavy metal toxicity and acidification-induced carbon destabilization) triggered by excessive gangue amendments [28]. The incorporation of microalgae similarly elicited a biphasic response in OM dynamics. However, comparative analysis revealed a significant reduction in OM content at equivalent coal gangue application rates relative to non-microalgae-amended treatments. This antagonistic interaction may be attributed to competitive substrate utilization, microbial metabolic shifts, or altered carbon stabilization pathways induced by the co-application of microalgae and coal gangue [29]. In this study, the dominant role is that algae promote the decomposition of organic matter in coal gangue through direct assimilation, absorption, and metabolism. Experimental treatments amended with 10% and 50% coal gangue exhibited a marked elevation in sulfate ion (SO_4_^2−^) concentration (Figure 2F), likely attributable to the oxidative weathering of sulfide minerals (e.g., pyrite) inherent in the gangue matrix. The amendment of microalgae significantly suppressed sulfate (SO_4_^2−^) accumulation in the soil system [30], potentially mediated through microbial–algal interactions such as biosorption of sulfate ions, enhanced assimilatory sulfate reduction by algal biomass, and/or competitive inhibition of sulfur-oxidizing bacterial activity [31].

### 3.2. Effect on Soil Enzyme Activities by Improvement Strategy

**Figure 3:**
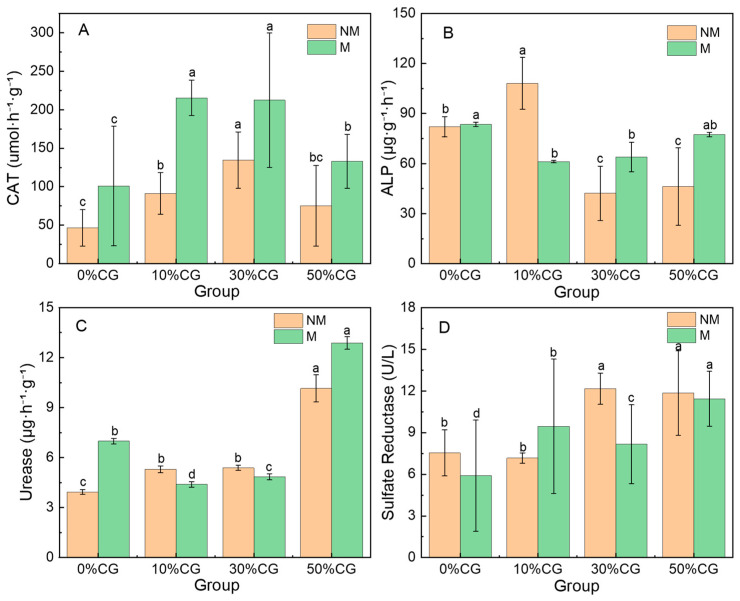
Enzyme activities varied during one year of incubation under different treatments. (**A**) Soil alkaline phosphatase; (**B**) soil catalase; (**C**) soil urease; (**D**) sulfate reductase. Error bars represent the standard deviation. Different letters indicate significant differences within the group (*p* < 0.05).

The effects of microalgae treatment (NM: no microalgae; M: with microalgae) on four soil enzyme activities across coal gangue (CG) addition ratios (0%, 10%, 30%, and 50%) are shown in Figure 3. As the proportion of coal gangue added changed, soil catalase (CAT) activity showed different trends under the two treatments (NM and M) (Figure 3A). Specifically, CAT activity demonstrated a hormetic dose–response relationship to incremental coal gangue application, characterized by a transient activation phase (≤30% gangue loading), followed by progressive enzymatic suppression at higher dosages (≥50% gangue loading). Notably, concomitant supplementation with microalgae significantly increased catalase activity (by 57.59%~136.01%) under equivalent gangue stress (*p* < 0.05). Consequently, microalgae supplementation mitigated the inhibitory effects of high coal gangue loading on CAT activity. Moreover, in the scenario of a low-proportion addition of coal gangue, microalgae was capable of facilitating an enhancement in CAT activity [23].

The amendment of coal gangue exhibits a dose-dependent modulation of ALP activity (Figure 3B), characterized by moderate enhancement at lower application rates (e.g., ≤10%) followed by significant inhibition under excessive dosages (e.g., ≥30%). The maximum ALP activity (108.1 ± 15.60 μg·g^−1^·h^−1^) was observed with 10% CG and was significantly higher than other treatment groups (*p* < 0.05), in contrast with the minimum value (46.2 ± 23.23 μg·g^−1^·h^−1^) with 50% CG. Microalgae amendment significantly enhances soil phosphatase activity under high-dose coal gangue stress (e.g., ≥30% *w/w*). Previous research has shown that adding 10% microalgae biomass can increase soil phosphatase activity by 30% to 50% [32].

The urease activity under M treatment was higher than that under NM treatment at 0% CG (Figure 3C). With increasing coal gangue addition ratios, the urease activity in NM treatments remained relatively stable across different proportions, showing minimal fluctuations. In contrast, M treatment exhibited a significant enhancement of urease activity at 50% CG (*p* < 0.05), which markedly exceeded both other proportions and NM treatments. This indicates that the interaction between microalgae and coal gangue substantially promotes urease activity under high coal gangue addition ratios.

The variations in sulfate reductase activity under different coal gangue addition ratios exhibited complex trends between the two treatments (Figure 3D). At a low coal gangue proportion (10% CG), the activities of both treatments remained relatively low. When the addition ratio increased to 30% CG, the activity of NM treatment peaked at its highest level, while M treatment showed a moderate increase. At 50% CG, NM treatment maintained its activity at a relatively high level, whereas M treatment displayed a slight decline but still retained comparatively elevated activity. These results suggest that microalgae partially influenced the response pattern of sulfate reductase activity to varying coal gangue addition ratios.

Both the addition ratio of coal gangue and the presence or absence of microalgae exert significant influences on the activities of CAT, ALP, urease, and SR in soil. This impact varies distinctly according to the enzyme type. Microalgae exhibit regulatory effects of different magnitudes on soil enzyme activities under diverse coal gangue addition ratios. Among them, they showed a significant enhancement effect on catalase activity, followed by an enhancement effect on phosphatase and urease activity, while they showed an inhibitory trend on sulfate reductase. In certain instances, they can mitigate the adverse effects of coal gangue on soil enzyme activities or contribute to the enhancement of enzyme activities [33].

### 3.3. Effect of Improvement Strategy on Microbial Community Structure and Functions

**Figure 4:**
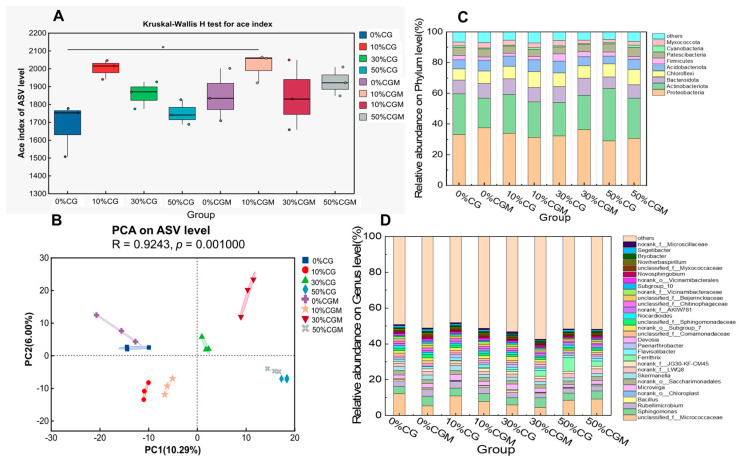
The composition of the microbial communities from different groups. (**A**) Alpha diversity analysis, the dot represents the sample, and the * represents *p* < 0.05; (**B**) Principal co-ordinate analysis with Bray–Curtis distance. (**C**) Microbial communities at the phylum level. (**D**) Microbial communities at the genus level.

The Ace index, a metric employed to assess microbial community richness, demonstrated distinct variations across experimental treatments in the present study. As illustrated in Figure 4A, the experimental data revealed a notable elevation in Ace index values across both coal gangue (CG) and coal gangue–microalgae co-treatment (CGM) groups at the 10% amendment level compared to other treatment conditions, indicating that the richness of soil microorganisms was also higher than other groups. Microalgal supplementation appears to amplify this concentration-dependent response pattern, particularly under elevated coal gangue amendment levels. Notably, the observed divergence in Ace index values between CG and CGM treatments suggests a potential modulatory effect of microalgal incorporation on community richness dynamics during coal gangue amendment. This pattern implies that microbial community assembly mechanisms may be differentially influenced by the presence or absence of microalgae across varying coal gangue addition gradients.

Principal component analysis (PCA) was employed to investigate *β*-diversity patterns in microbial community structure across experimental treatments. The ordination plot (Figure 4B) demonstrates the sample distribution along two primary axes, with principal component 1 (PC1, abscissa) accounting for 10.29% of the total variance and principal component 2 (PC2, ordinate) explaining an additional 6.94% of compositional variation. Distinct chromatic and geometric symbols represent sample clusters from discrete treatment groups, revealing spatial separation patterns that reflect treatment-specific microbial consortia configurations. The limited cumulative explanatory power (17.23%) of the first two principal components suggests substantial high-dimensional heterogeneity in community assembly processes, potentially attributable to multifactorial interactions between coal gangue gradients, microalgal supplementation, and inherent soil biogeochemical parameters.

In the microbial community analysis, 33 OTUs were observed at the phylum level, and the phylum-dominant patterns were consistent across all eight experimental groups, mainly including Proteobacteria, Actinobacteria, Bacteroidota, Chloroflex, Acidobacteriota, Firmicutes, Patescibacteria, Cyanobacteria, and Myxococcota. Among them, Proteobacteria and Actinobacteria collectively constituted over 60% of the relative abundance in each treatment (Figure 4C). Notably, Proteobacteria demonstrated an initial relative abundance of approximately 30% in both control groups (0% CG and 0% CGM), exhibiting a non-linear response to coal gangue gradients. Their abundance followed a biphasic trajectory characterized by an initial decline (10–30% CG/CGM), subsequent recovery (30–50% CG), and eventual reduction (50% CGM), suggesting concentration-dependent metabolic adaptation thresholds. In contrast, Actinobacteria displayed a marked progressive increase along the coal gangue gradient, ascending from 30% in control groups to nearly 50% under high-amendment conditions (50% CG/CGM). This divergent response pattern between the two dominant phyla implies distinct ecological strategies under environmental stress gradients: while Proteobacteria populations appear sensitive to intermediate amendment levels, Actinobacteria demonstrated robust competitive dominance under elevated coal gangue exposure.

Microbial phylogenetic composition analysis demonstrated remarkable stability in genus-level community structure across all experimental treatments. In this study, a total of 1019 OTUs were observed at the genus level. As evidenced in Figure 4D, *uncultured*_*f*_*Micrococcaceae* maintained overwhelming dominance throughout the experimental matrix, constituting 6.99–18.98% of relative abundance across treatment groups. The abundance of this species decreased in the group with added microalgae, compared to the group without added microalgae. In addition, the bacterial genus *Sphingomonas* demonstrated significant ecological advantages, exhibiting a progressive increase in relative abundance following microalgal supplementation. This observed proliferation may be attributed to the genus’ well-documented metabolic capabilities in xenobiotic degradation, as evidenced by prior research demonstrating its efficacy in breaking down various organic contaminants including monocyclic aromatic hydrocarbons, biphenyl derivatives, substituted aromatic compounds, and polycyclic aromatic hydrocarbons (PAHs) [34]. This result will be beneficial for the degradation of recalcitrant organic compounds in coal gangue, providing new ideas for the green use of coal gangue.

The incorporation of coal gangue exerts a significant modulatory influence on the structural complexity, compositional profile, and biodiversity indices of indigenous soil microbial consortia, with discernible variations observable across multiple taxonomic hierarchies. These alterations manifest as hierarchical modifications spanning phylum-level phylogenetic distributions to genus-specific abundance patterns. Previous studies have also demonstrated that coal gangue provided a favorable habitat for microorganisms, increasing their population and activity [35]. The observed ecological modifications are mechanistically attributable to synergistic interactions between the inherent physicochemical characteristics of coal gangue and subsequent pedospheric environmental modifications (encompassing redox potential fluctuations, nutrient cycling alterations, and microhabitat restructuring) [9].

**Figure 5:**
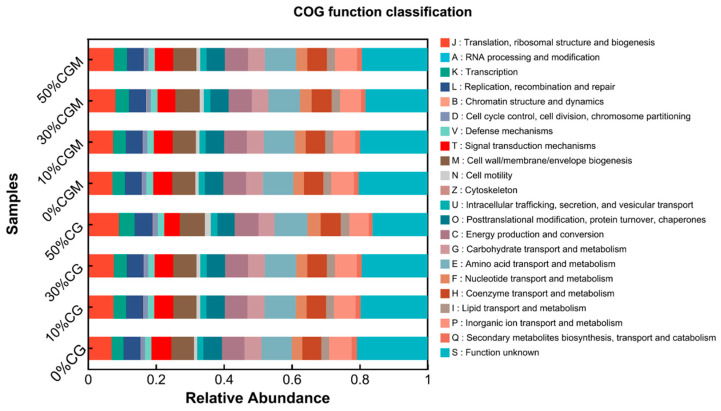
Differences in microbial communities and functional prediction among different treatments.

A systematic survey was conducted to depict the distribution of typical functional genes of microorganisms in CG and CGM samples under different concentration gradients (Figure 5). Commonly, some COG functional categories, such as “Translation, ribosomal structure and biogenesis” (J), exhibit relatively high abundances in most samples, highlighting their significance. This indicates that microorganisms are more active in protein synthesis-related functions, which are fundamental for maintaining normal microbial growth and metabolism [36]. The relative abundance differences of this functional category between different treatment groups may affect the growth rate and metabolic efficiency of microorganisms. As the proportion of CGM or CG addition increases from 0% to 50%, the proportion of some functional categories changes. For example, the relative abundance of functional categories that may be related to substance transport and metabolism (such as E: amino acid transport and metabolism, and G: carbohydrate transport and metabolism) fluctuates in samples with different addition ratios, suggesting that the added components have an impact on the metabolic pathways of substances in organisms [37].

### 3.4. Growth Status of Plants in Different Groups

*Medicago sativa* L., as one of the principal forage grasses in Inner Mongolia, exhibits notable characteristics of wide distribution and high yield. Cultivating *Medicago sativa* L. in soils of reclaimed mining areas effectively creates new pathways for the sustainable development of the local livestock industry.

**Figure 6:**
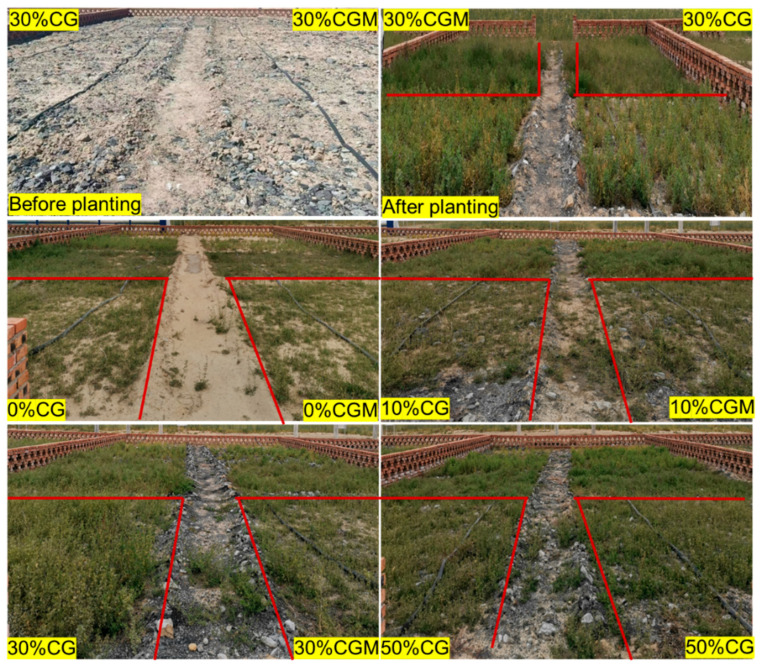
Growth status of *Medicago sativa* L. in different treatment groups (the red block diagram indicates the alfalfa planting area).

After one year of planting, the growth of alfalfa is shown in Figure 6. Prior to seeding interventions, the substrate matrix amended with 30% coal gangue (*w/w*) manifested as a heterogeneous particulate system characterized by gravel, siliceous sand, and coal gangue fragments, maintained its unmanaged pedogenic configuration. The experimental plots remained in a non-agriculturalized state with minimal surface horizon development. Post-implantation observations confirmed successful seedling establishment of *Medicago sativa* L. across all treatment modalities, demonstrating complete plot coverage irrespective of substrate composition variations. In the experimental groups without CG and CGM amendments (0% CG and 0% CGM treatments), *Medicago sativa* L. demonstrated significantly lower vegetative coverage compared to amended groups. The *Medicago sativa* L. coverage of the 30% CG and 30% CGM groups was significantly higher than that of the low-proportion addition group, and the plant growth was more lush, with good distribution uniformity. This indicated that adding 30% CG or CGM could significantly improve the growth environment of *Medicago sativa L.*, promoting its growth and development, and enhancing community coverage and the plant health status.

Overall, the addition of CG and CGM had a promoting effect on the growth of *Medicago sativa* L., and within a certain concentration range, the improvement trend of *Medicago sativa*’s L. growth status was obvious with the increase of the addition ratio. However, whether there is still a positive effect beyond a certain range needs further research and verification. In addition, we also found that the application of CG and CGM induced significant phenological changes in *Medicago sativa* L. Compared with the unmodified group, the modified group extended the pre-productive stage by 19–24 days and reduced the aging progression rate by 32–41%. The CGM group made this effect more pronounced, possibly because microalgae components may specifically enhance photoperiod plasticity through auxin-mediated pathways, demonstrating their critical role in chronobiological regulation [38].

**Figure 7:**
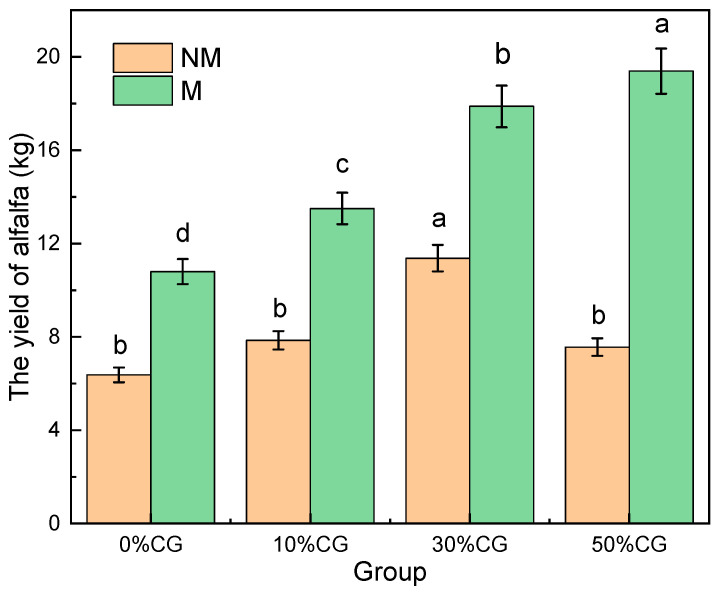
Biomass of *Medicago sativa* L. in different treatment groups. Different letters indicate significant differences within the group (*p* < 0.05).

In addition, this study also investigated the biomass of annual *Medicago sativa* L. under different treatments (Figure 7). The biomass of *Medicago sativa* L. showed a trend of first increasing and then decreasing with the increase of coal gangue material addition, reaching its maximum value when 30% was added, significantly higher than other treatment groups (*p* < 0.05). Similarly, the addition of microalgae significantly increased the growth of *Medicago sativa* L., especially in the group with 50% coal gangue material added, and there were significant differences between different treatments (*p* < 0.05). These agronomic enhancements were concomitant with CG-induced pedostructural modifications, particularly in soil porosity and micronutrient mobilization, demonstrating its efficacy as a growth-mediating substrate [39].

### 3.5. Mechanism of Soil Improvement by Coupling the Coal Gangue Ecological Matrix with Microalgae

**Figure 8:**
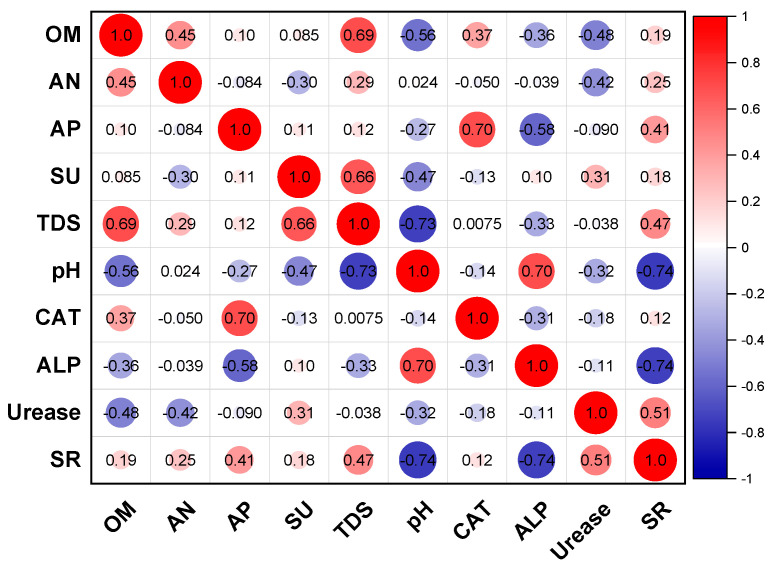
Pearson correlation analysis of soil physicochemical properties and enzyme activity. OM: organic matter, AN: available nitrogen, AP: available phosphorus, SU: sulfate, TDS: total dissolved solids, CAT: catalase, ALP: alkaline phosphatase, SR: sulfate reductase.

The heatmap illustrates correlations between multiple environmental variables and soil enzyme activity (Figure 8). The results demonstrate a significant positive correlation (*r* = 0.37) between OM and CAT. This association may stem from the role of organic matter in providing carbon sources and essential nutrients for enzyme-producing microorganisms, thereby regulating biosynthetic processes that modulate enzyme activity [40,41]. AN and urease showed a strong negative correlation (coefficient −0.42), which is consistent with the functional characteristics of urease participating in nitrogen cycling, decomposing urea into ammonia to increase soil alkaline hydrolyzable nitrogen content, and the two’ interact with each other. The analysis revealed a statistically significant positive correlation between AP and CAT activities (coefficient 0.70), whereas ALP exhibited a marginally negative association with CAT (coefficient −0.56). This contrasting pattern suggests distinct regulatory mechanisms: AP–CAT synergy may stem from shared biogeochemical drivers (e.g., organic matter mineralization), while the negligible ALP–CAT relationship could reflect functional decoupling in phosphorus and redox metabolic pathways [42]. These differential associations align with prior findings that AP primarily mediates organic phosphorus hydrolysis under acidic conditions [43], whereas ALP dominates in alkaline environments, potentially explaining its limited interaction with CAT in this study’s pH range (5.8–6.3). In addition, a statistically significant positive correlation was showed between soil pH and catalase (CAT) activity (coefficient 0.70), while a significant negative correlation was observed with SR activity (coefficient −0.74). This antagonistic relationship suggests pH-dependent regulatory divergence: alkaline conditions may stabilize CAT structure or enhance its microbial production, whereas acidic shifts likely inhibit SR-mediated sulfate reduction processes, potentially through redox potential constraints or the suppression of sulfate-reducing bacterial communities. These results align with SR’s sensitivity to pH-driven redox fluctuations—acidic conditions (pH < 6.0) typically suppress sulfate-reducing bacteria (e.g., *Desulfovibrio* spp.) by altering electron donor availability [44], whereas neutral-alkaline pH favors CAT preservation via reduced oxidative denaturation [45].

**Figure 9:**
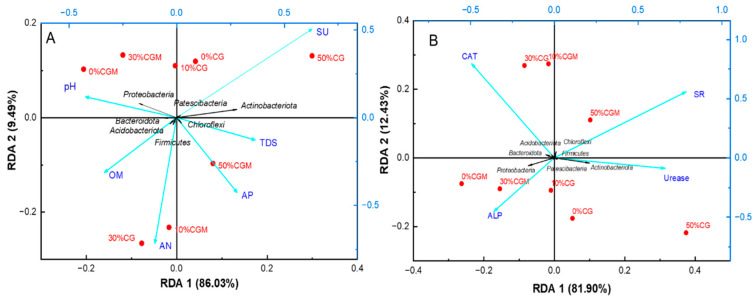
Relationship between soil microbial community and physicochemical characteristics variables (**A**), enzyme activities (**B**).

Redundancy analysis (RDA) was implemented to quantify the multivariate relationships between environmental variables and microbial community composition. The ordination model revealed two statistically significant canonical axes, with RDA1 explaining 86.03% of the constrained variance and RDA2 accounting for an additional 9.49% (cumulative explanation: 95.52%) (Figure 9A). Microbial communities were closely correlated with soil physical and chemical properties. The red dot of representing CGM is closer to the green blue of representing pH, AP, and AN. The results showed that the addition of algae was more likely to affect changes in soil pH and nutrient content, under the same proportion of coal gangue addition. There is a significant positive correlation between pH with Proteobacteria and Bacteroidota Acidobacteriota, and a significant negative correlation with Patescibacteria, Actinobacteriota, and Choloroflexi. The AP value had a significant positive correlation with Choloroflexi and Firmicutes, and a significant negative correlation with Proteobacteria and *Bacteroidota Acidobacteriota*. The relationship between AN and these microorganisms was similar to that of AP. Therefore, under certain pH conditions, it was more favorable for Choloroflexi and Firmicutes to release available phosphorus and available nitrogen. In addition, it was worth noting that high levels of coal gangue (50%) may lead to an increase in soil sulfate content, but this increase can also be alleviated by the addition of microalgae, which further proved that the combination of coal gangue and microalgae is more conducive to improving soil ecology.

The relationship between soil enzyme activities and microbial communities revealed a cumulative variance explanation of 94.33% for the two ordination axes (Figure 9B). Compared to the coal gangue group without microalgae amendment, microalgae addition significantly enhanced the activity of all four enzymes.

APL was significantly positively correlated with the major functional phyla Proteobacteria and Bacteroidota, and significantly negatively correlated with Actinobactenota. The relationship between CAT and these three phyla mirrored that of ALP, indicating synchronized microbial influence on both enzymes.

In addition, the abundance of Proteobacteria and Bacteroidota increased after microalgae were added, which also means that microalgae amendment improves the soil environment and enhances ALP and CAT activity. It is worth noting that adding 50% CG and 50% CGM to the group may increase the activity of soil sulfate reductase and urease. This likely occurs because coal gangue contains abundant sulfate, which serves as a substrate for sulfate reductase and directly stimulates enzyme activity [46]. Consequently, these findings underscore the importance of controlling coal gangue application rates during resource utilization.

**Figure 10:**
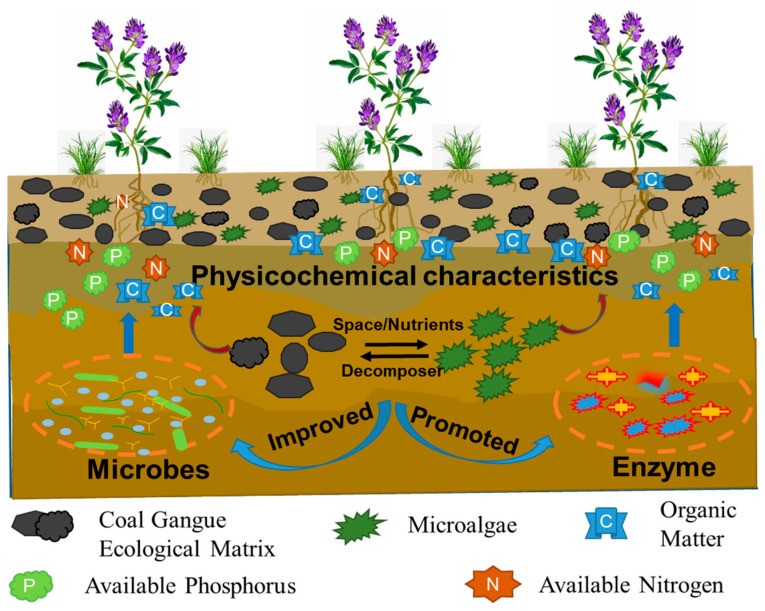
Schematics of the biological improvement system and its application for ecological restoration in mining areas.

Based on the above discussions, the mechanism of soil improvement by a coal gangue ecological matrix coupled with microalgae is proposed (Figure 10). Microalgae are crucial for coal gangue-based ecological materials. Coal gangue provides a habitat and nutrients for microalgae and microorganisms, while microalgae and microorganisms decompose coal gangue. The proposed approach demonstrates the dual synergistic effects in the coal gangue ecological matrix coupled with microalgae improvement. Firstly, through metabolic coupling with soil microbial consortia, it significantly enhances the bioconversion efficiency of recalcitrant carbon, nitrogen, and phosphorus fractions in coal gangue matrices. This is achieved primarily by improving the activity of key soil enzymes, specifically alkaline phosphatase and urease. Secondly, it mitigates coal gangue-induced abiotic stressors (e.g., polycyclic aromatic hydrocarbons, heavy metals, and sulfate) via stimulating antioxidant enzyme biosynthesis (CAT activity increased by 57.93%~136.01%) and maintaining functional enzyme stability (urease activity elevated by 19.38%). Moreover, we can conclude that controlling the amount of coal gangue matrix added within 30%, combined with the ecological material formed by microalgae addition, represents an optimal solution for soil improvement.

## 4. Conclusions

In conclusion, this study demonstrates the potential of coupling a coal gangue ecological matrix with microalgae for soil improvement in mining areas. Field experiments at Jintong Coal Mine revealed that microalgae significantly mitigate the negative impacts of coal gangue on soil properties, including acidification and elevated total dissolved solids The combination of the coal gangue addition with microalgae not only promoted *Medicago sativa* L. growth, but also but also improved the nutrient cycling and ecological functions of the soil, highlighting its potential for large-scale application. This approach provides a sustainable solution for soil improvement and ecological restoration in coal mining areas, suggesting that proper management of coal gangue and utilization of microalgae can turn waste into a valuable resource for environmental improvement. However, this study currently only focuses on the changes in soil properties and alfalfa growth within a one-year cycle, which has certain limitations for the deep analysis of coal gangue and microalgae in soil improvement mechanisms. In the future, we need to pay long-term attention to the impact of these measures on soil ecology.

## Figures and Tables

**Table 1:** Experimental design.

Group	Primitive Soil		Soil Containing 10% Coal Gangue		Soil Containing 30% Coal Gangue		Soil Containing 50% Coal Gangue	
	NM	M	NM	M	NM	M	NM	M
**Group Name**	0% CG	0% CGM	10% CG	10% CGM	30% CG	30% CGM	50% CG	50% CGM

## Data Availability

The original contributions presented in this study are included in this article; further inquiries can be directed to the corresponding authors.
